# Live-attenuated ME49Δ*cdpk3* strain of *Toxoplasma gondii* protects against acute and chronic toxoplasmosis

**DOI:** 10.1038/s41541-022-00518-5

**Published:** 2022-08-20

**Authors:** Minmin Wu, Shutong Liu, Ying Chen, Deng Liu, Ran An, Haijian Cai, Jie Wang, Nan Zhou, Cudjoe Obed, Meng Han, Jilong Shen, Lijian Chen, Jian Du

**Affiliations:** 1 Department of Biochemistry and Molecular Biology, School of Basic Medical Sciences, Anhui Medical University, Hefei, China; 2 The Research Center for Infectious Diseases, School of Basic Medical Sciences, Anhui Medical University, Hefei, China; 3 The Provincial Key Laboratory of Zoonoses of High Institutions of Anhui, Anhui Medical University, Hefei, China; 4 The Key Laboratory of Microbiology and Parasitology of Anhui Province, Anhui Medical University, Hefei, China; 5 Department of Anesthesiology, The First Affiliated Hospital of Anhui Medical University, Hefei, China

**Keywords:** Live attenuated vaccines, Live attenuated vaccines, Infection

## Abstract

Toxoplasmosis, a common parasitic disease, is caused by *Toxoplasma gondii*, which infects approximately 30% of the world’s population. This obligate intracellular protozoan causes significant economic losses and poses serious public health challenges worldwide. However, the development of an effective toxoplasmosis vaccine in humans remains a challenge to date. In this study, we observed that the knockout of calcium-dependent protein kinase 3 (CDPK3) in the type II ME49 strain greatly attenuated virulence in mice and significantly reduced cyst formation. Hence, we evaluated the protective immunity of ME49Δ*cdpk3* as a live attenuated vaccine against toxoplasmosis. Our results showed that ME49Δ*cdpk3* vaccination triggered a strong immune response marked by significantly elevated proinflammatory cytokine levels, such as IFN-γ, IL-12, and TNF-α, and increased the percentage of CD4^+^ and CD8^+^ T-lymphocytes. The high level of *Toxoplasma*-specific IgG was maintained, with mixed IgG1/IgG2a levels. Mice vaccinated with ME49Δ*cdpk3* were efficiently protected against the tachyzoites of a variety of wild-type strains, including type I RH, type II ME49, Chinese 1 WH3 and Chinese 1 WH6, as well as the cysts of wild-type strains ME49 and WH6. These data demonstrated that ME49Δ*cdpk3* inoculation induced effective cellular and humoral immune responses against acute and chronic *Toxoplasma* infections with various strains and was a potential candidate to develop a vaccine against toxoplasmosis.

## Introduction

*Toxoplasma gondii* (*T. gondii*) is a common zoonotic intracellular parasite capable of infecting almost all warm-blooded animals, including humans^1^. Approximately 30% of the world’s population is infected with *T. gondii*; most infections are asymptomatic cases in healthy individuals, but infection in immunocompromised patients and developing fetuses of pregnant women can cause severe disease and death^2–4^. In addition, primary infection in many important agricultural animals, such as goats, leads to abortion and stillbirth, as well as major economic losses and severe challenges in the livestock industry^3,5^. In animals and humans, the fixed combination of pyrimethamine and sulfadiazine, which targets tachyzoites, is the current standard treatment for active toxoplasmosis; however, it has been proven ineffective against bradyzoites and the latent stage of the infection^6,7^.

*T. gondii* has a complicated life cycle and multiple transmission routes. Domestic and other wild cats are the definitive hosts of *T. gondii*, and ingestion of oocysts excreted by cats is the main source of infection in intermediate hosts (humans and animals)^1,8^. In addition, *T. gondii* can spread among intermediate hosts through asexual reproduction and predation^3^. *Toxoplasma* strains have a complex population structure. There are three main types of *Toxoplasma strains* (I, II, and III) in North America and Europe. The main type I strains are RH and GT1, the main type II strains are PRU and ME49, and the main type III strain is CEP, and their virulence varies greatly^9–11^. *Toxoplasma* strains in South America are more genetically diverse than those in North America and Europe. However, genotype Chinese 1 has been identified as the predominant strain in East Asia, particularly in China^12,13^. Jensen and collaborators showed that strains from different genotypes can infect the same host repeatedly^14^. The above characteristics of *Toxoplasma* strains pose great challenges to the control of toxoplasmosis. Thus, the development of an effective toxoplasmosis vaccine is critical for limiting the infection of various *Toxoplasma* strains.

In recent years, several studies aimed at developing a safe and effective *T. gondii* vaccine have been conducted. Nucleic acid vaccines^15,16^, recombinant protein vaccines^17–19^ and cocktail antigen vaccines^20^ have been developed to prevent *T. gondii* infection; however, none have provided adequate protection. To date, the most promising strategy for obtaining an efficient *Toxoplasma* vaccine is the use of live attenuated vaccines, which can induce higher and long-term protective cellular and humoral immune responses to prevent *Toxoplasma* infection^21–23^. Currently, Toxovax^®^ is the only commercially available live attenuated S48 strain vaccine, and it is only licensed for preventing congenital toxoplasmosis in sheep^24,25^.

*T. gondii* possesses 14 genes that code for Ca^2+^-dependent protein kinases (CDPKs), which are involved in motility, invasion, replication, and egress^26–28^. *Tg*CDPK3 is essential for the rapid induction of parasite egress and the establishment of chronic infection in mice, and it is the key to the virulence of the parasite in vivo^29,30^. In the current study, knockout of the CDPK3 gene in the type II ME49 strain severely attenuated the virulence of mice. Importantly, immunization of *BALB/c* mice with ME49Δ*cdpk3* induced effective protection against different parasite strains. Therefore, we evaluated ME49Δ*cdpk3* as a potential vaccine candidate against *Toxoplasma* infection.

## Results

### Knockout of CDPK3 in the type II ME49 strain severely attenuated virulence in mice

**Fig. 1:**
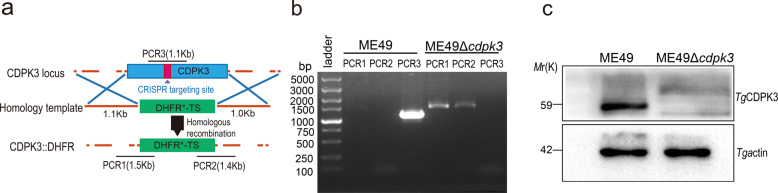
CRISPR/Cas9-mediated generation of CDPK3 mutant in *Toxoplasma gondii* Type II ME49 strain. **a** Schematic of CRISPR/CAS9 strategy for replacing CDPK3 with pyrimethamine-resistant DHFR (DHFR*-TS) and PCR identification of ME49Δ*cdpk3* clones. **b** Diagnostic PCRs for the mutant of ME49Δ*cdpk3 T. gondii* strain. PCR1 and PCR2 detect for 5’and 3’integration of the selection marker, while PCR3 checks for successful deletion of the CDPK3 gene. Parental wild-type ME49 strain (WT) was used as a control. **c** Western blotting for detection of CDPK3 expression in ME49 WT and ME49Δ*cdpk3*. *T. gondii* actin antibody was included as a loading control. All blots derive from the same experiment and were processed in parallel.

**Fig. 2:**
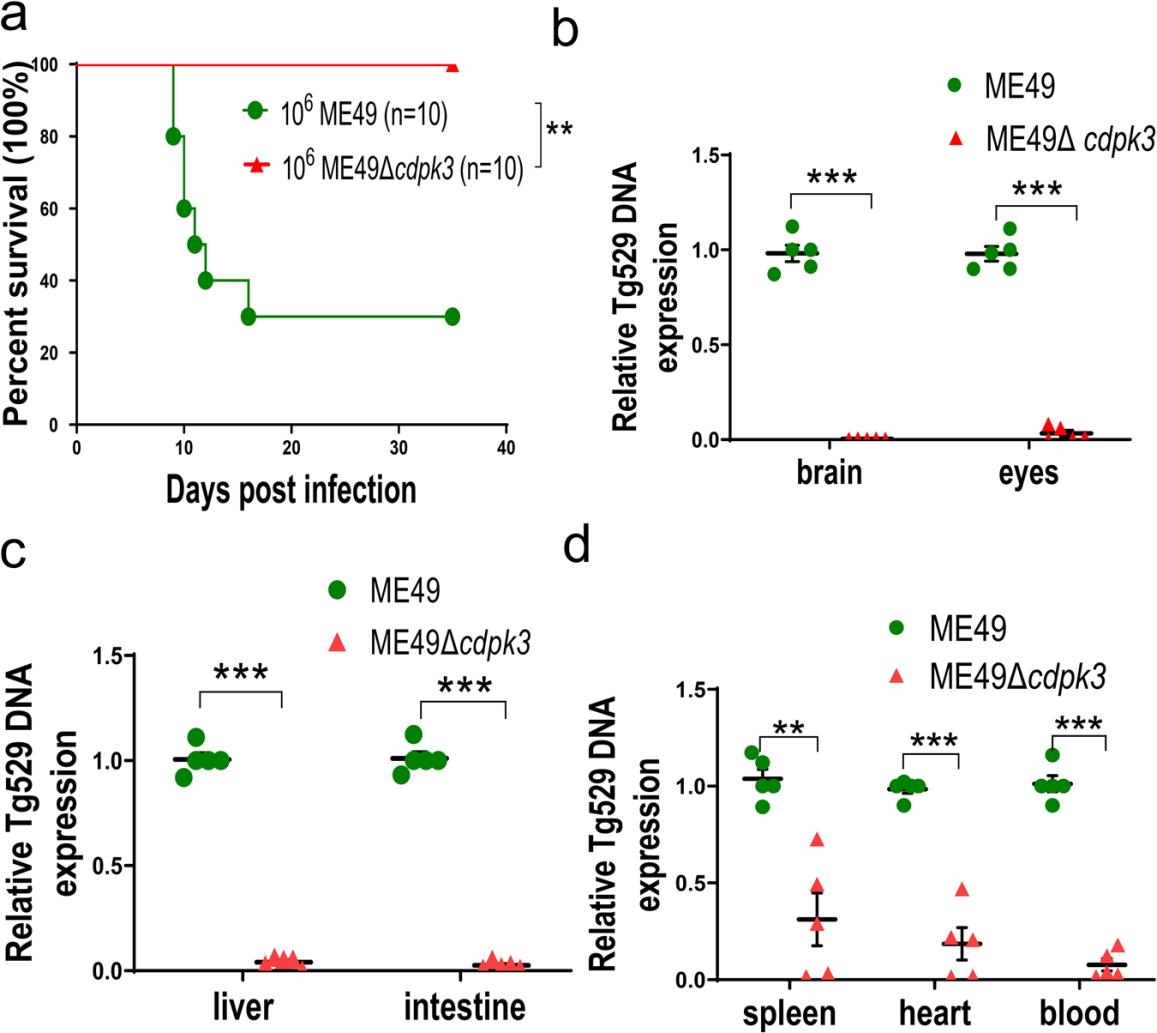
The mutant of CDPK3 in ME49 strain severely attenuated the virulence in mice. Female *BALB/c* mice were infected with 10^6^ wild-type or ME49Δ*cdpk3* parasites by intraperitoneal injection. **a** Survival rate of mice was monitored for 35days (*n* = 10 per group). **p < 0.01. Gehan–Breslow–Wilcoxon tests. **b**–**d** Seven-days post-infection, parasite burdens in brain, eyes, liver, ileum, spleen, heart, and blood were estimated by quantitative PCR (*n* = 5 each group). All statistical analyses were performed by using unpaired t test. Bars = mean ± standard error of the mean (SEM). Statistical differences are represented by ***p < 0.001, **p < 0.01.

To determine whether CDPK3-deficient ME49 tachyzoites may be employed as a live-attenuated vaccine against acute and latent *T. gondii* infection in mice, we knocked out the CDPK3 gene. The CDPK3 coding sequence was deleted using CRISPR/Cas9 technology (Fig. 1a). The sgRNA and DHFR*-TS markers were inserted into the CDPK3 coding sequence, and western blotting and PCR results showed that CDPK3-deficient ME49 tachyzoites were successfully generated (Fig. 1b, c and Supplementary Fig. 1). *BALB/c* mice were intraperitoneally infected with 10^3^, 10^4^, 10^5^, and 10^6^ tachyzoites of the parental ME49 strain or the CDPK3 knockout ME49 strain to assess the effect of CDPK3 inactivation on parasite virulence. Subsequently, the survival percentage of mice was monitored for 35 days postinfection. The results showed that the virulence of ME49Δ*cdpk3* tachyzoites was significantly attenuated, and the survival rate was 100% even at an infectious dose of 10^6^ tachyzoites compared with the wild-type ME49 strain (Fig. 2a). Tissue samples were obtained from challenged mice at 7 dpi to evaluate parasite burden. ME49Δ*cdpk3* caused a significant reduction in parasite burden (Fig. 2b–d). To test the potential of this mutant strain as a good vaccine and the immunogenicity derived from ME49Δ*cdpk3* vaccination, mice were infected with different doses of parasites, including 10^3^, 10^4^, 10^5^, and 10^6^, and the mice were observed for clinical symptoms for 35 days. Our preliminary experiments showed that a low clinical score was observed in 10^3^, 10^4^ and 10^5^ ME49Δ*cdpk3-*infected mice, whereas 10^6^ ME49Δ*cdpk3*-infected mice exhibited slight clinical signs, and mice infected with different doses of wild-type ME49 showed severe clinical signs (Supplementary Fig. 2a). Next, although we did observe that mice infected with ME49Δ*cdpk3* tachyzoites formed brain cysts, the cyst number in the brains of mice infected with ME49Δ*cdpk3* was significantly reduced compared with that in the brains of mice infected with wild-type ME49 (Supplementary Fig. 2b). In addition, serum from the above-infected mice was collected on Day 35 post-infection, and the levels of *Toxoplasma*-specific IgG were determined. *T. gondii*-specific IgG in the sera of 10^3^, 10^4^, 10^5^ and 10^6^ ME49Δ*cdpk3*- and 10^3^, 10^4^ and 10^5^ wild-type ME49-infected mice were of similarly high levels (Supplementary Fig. 3). Therefore, an infectious dose of 10^3^ ME49Δ*cdpk3* tachyzoites was selected for the subsequent vaccination experiment (Supplementary Fig. 4).

### ME49Δ*cdpk3* vaccination confers protection against acute infection with various tachyzoites

**Fig. 3:**
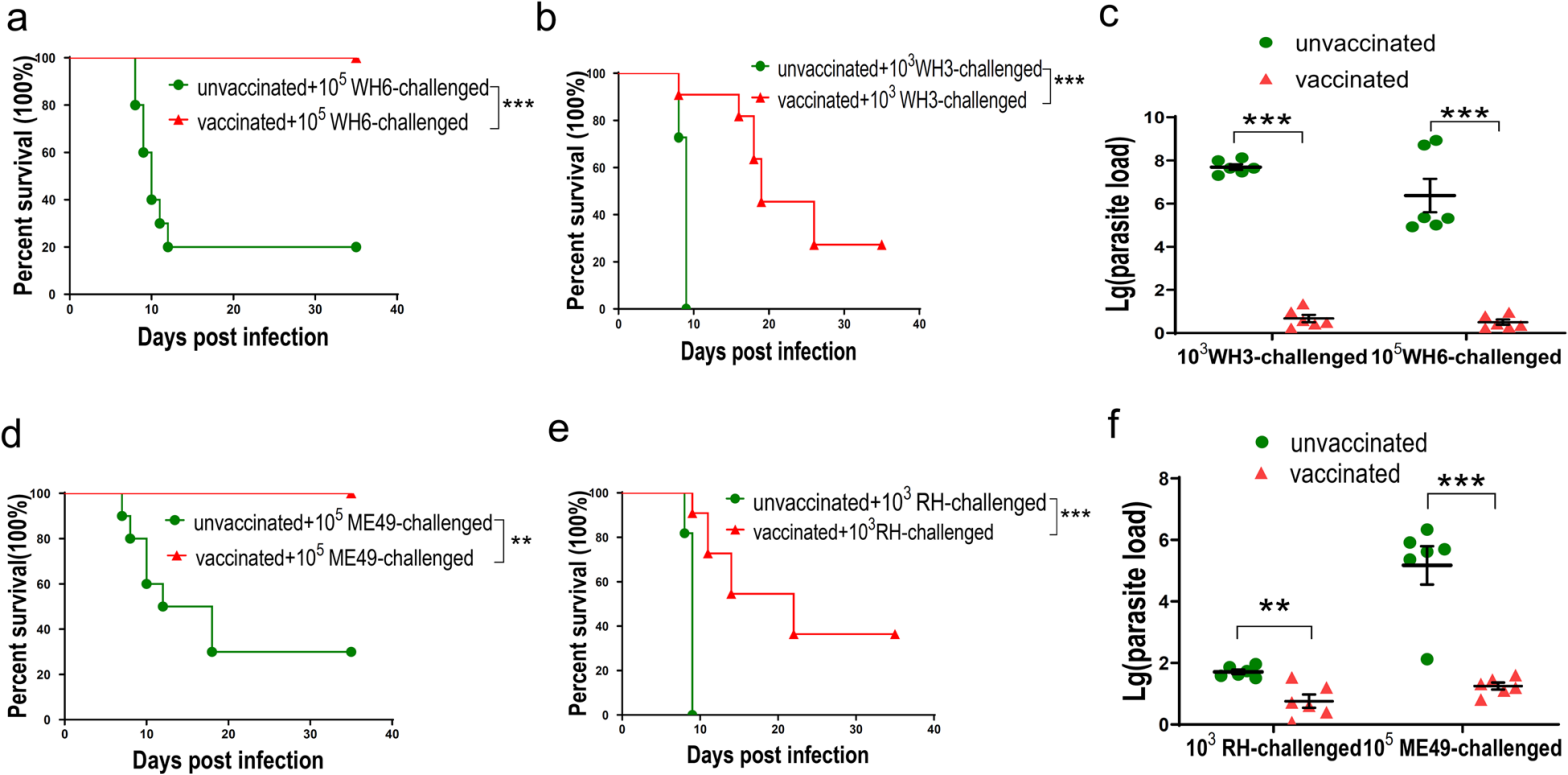
Immunization with ME49Δ*cdpk3* vaccine protects mice from *Toxoplasma* tachyzoite infection. *BALB/c* mice were pre-immunized with 10^3^ ME49Δ*cdpk3* tachyzoites. **a**, **b** 75 days after vaccination, they were respectively infected with **a** 10^5^ WH6 strain tachyzoites, **b** 10^3^ WH3 strain tachyzoites by intraperitoneal injection and their mortality was recorded for additional 35 days (10 mice for each strain). **d**–**e** 125 days after vaccination, they were respectively challenged with **d** 10^5^ tachyzoites of the ME49 strains, **e** 10^3^ tachyzoites of the RH strains by intraperitoneal injection and their mortality was recorded for additional 35 days (10 mice for each strain). Nonimmunized mice were considered as negative control. **p < 0.01, ***p < 0.001. Gehan-Breslow-Wilcoxon tests. **c**, **f** Parasite loads in the peritoneal fluid of vaccinated mice challenged with WH3, WH6, RH or ME49 tachyzoites. A quantitative PCR was used to determine parasites burdens in vaccinated and unvaccinated challenged mice at 7 days post-infection (*n* = 6 each group). All statistical analyses were performed by using unpaired t test. Bars = mean ± SEM. Statistical differences are represented by ***p < 0.001, **p < 0.01.

To assess the protective efficacy of ME49Δ*cdpk3* immunization against tachyzoite infection, *BALB/c* mice were vaccinated with 10^3^ ME49Δ*cdpk3* tachyzoites, and then the vaccinated mice were challenged with 10^3^ Chinese 1 strain WH3 or 10^5^ WH6 at 75 dpv. The survival rate was 100% in vaccinated mice infected with the less virulent WH6 strain, while the survival rate was only 20% in unvaccinated mice (Fig. 3a). However, when challenged with the virulent WH3 strain, although only 30% of vaccinated mice survived, the survival time was significantly extended in the vaccinated group (Fig. 3b). To check whether ME49Δ*cdpk*3 vaccination provides long-term protective immunity, the vaccinated mice were challenged with 10^3^ type I (RH) or 10^5^ type II (ME49) at 125 dpv. The results showed that no mortality was observed in immunized mice challenged with ME49 tachyzoites (Fig. 3d). The survival rate of vaccinated mice rechallenged with RH tachyzoites was also extended compared with that of unvaccinated mice (Fig. 3e). To further understand why ME49Δ*cdpk3* vaccination protects mice from acute *T. gondii* infections, peritoneal fluid and sera from reinfected ME49Δ*cdpk3*-vaccinated mice were collected at 7 days post-infection to assess parasite load. In unvaccinated mice, RH, ME49 and Chinese 1 WH3 and WH6 infections led to rapid parasite proliferation. However, we detected very few parasites in vaccinated mice challenged with these various strains (Fig. 3c, f), suggesting that the vaccination promotes rapid elimination of infecting parasites. Therefore, these results indicate that the live attenuated ME49Δ*cdpk3* vaccination confers longer-lasting and stronger protection against less virulent parasite strains but weaker protection against virulent strains.

**Fig. 4:**
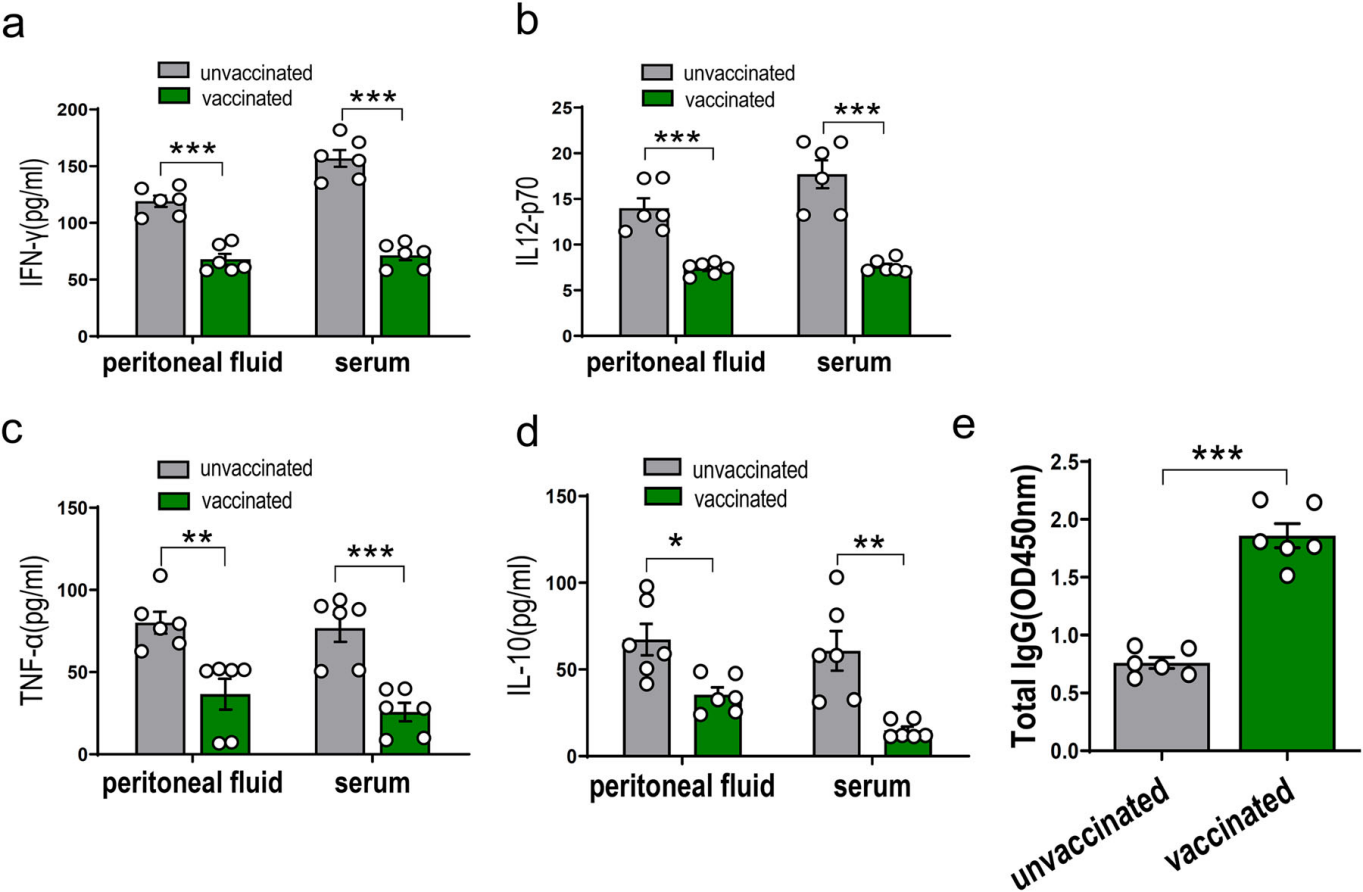
Cytokine and *T. gondii* specific IgG of the vaccinated or un-vaccinated mice challenged with WH3 strain. Cytokine and *T. gondii* specific IgG in the serum and peritoneal fluid of the vaccinated or un-vaccinated mice infected with Chinese1 WH3 strain were determined at 7 dpi. **a** IFN-γ, **b** IL-12p70, **c** TNF-α or **d** IL-10 levels in serum or peritoneal fluid. **e** The levels of *T. gondii* specific IgG in serum samples (*n* = 6 each group). All statistical analyses were performed by using unpaired t test. Bars = mean ± SEM. Statistical differences are represented by ***p < 0.001, **p < 0.01, *p < 0.05.

Additionally, the cytokine levels of ME49Δ*cdpk3*-vaccinated mice challenged with the Chinese 1 WH3 strain were also determined. The levels of IFN-γ, IL-12p70, TNF-α and IL-10 in both peritoneal fluid and sera were significantly decreased in the vaccinated mice compared with those of unvaccinated mice (Fig. 4a–d). More importantly, the *T. gondii*-specific IgG level remained high in all vaccinated mice (Fig. 4e). The results revealed that vaccination with ME49Δ*cdpk3* downregulates the severe inflammatory response during acute *Toxoplasma* infection and induces the humoral response. Taken together, ME49Δ*cdpk3* vaccination mounts an effective and safe protective immunity against challenging parasites, such as the classical North American and European strains and the predominant genotype of China.

### ME49Δ*cdpk3* vaccination confers protection against chronic infection

**Fig. 5:**
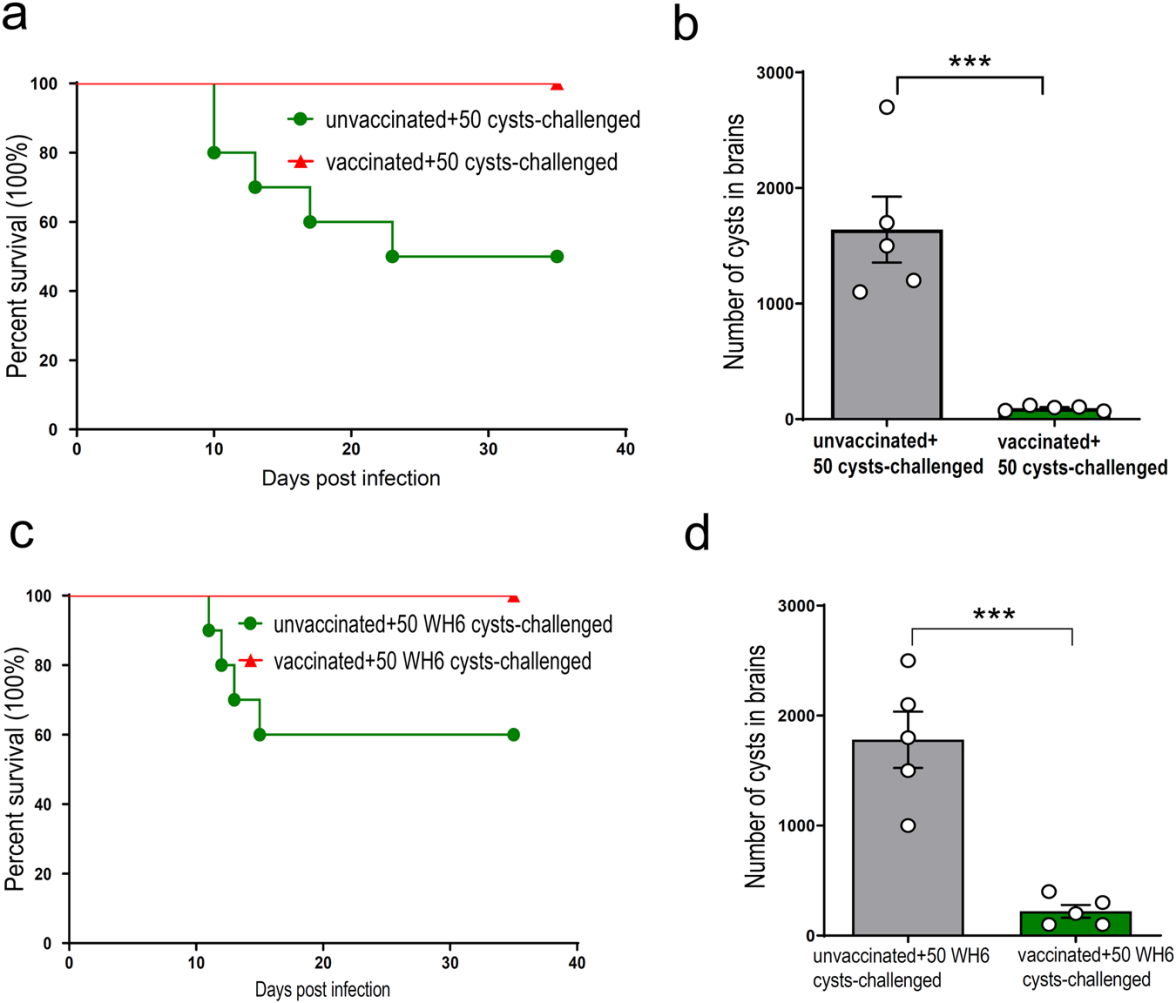
ME49Δ*cdpk3* vaccination protected mice against different cysts infection. **a** The survival rate of vaccinated or unvaccinated mice with 50 cysts of ME49 strain was monitored (*n* = 10 each group). **b** At 35 days post-infection with ME49 cysts, surviving unimmunized and immunized mouse brains were isolated to assess the number of cysts (*n* = 5 each group). The statistical analysis was performed by using unpaired t test. Bars = mean ± SEM. ***p < 0.001. **c** The survival rate of vaccinated or unvaccinated mice with 50 cysts of WH6 strain was monitored (*n* = 10 each group). **d** At 35 days post-infection with WH6 cysts, surviving unimmunized and immunized mouse brains were isolated to assess the number of cysts (*n* = 5 each group). The statistical analysis was performed by using unpaired t test. Bars = mean ± SEM. ***p < 0.001.

To assess the protective efficacy of the ME49Δ*cdpk3* vaccine against chronic infection, the mice were orally infected with 50 cysts of the ME49 or WH6 strain at 75 days after vaccination. The results showed that the survival rate in ME49Δ*cdpk3*-vaccinated mice challenged with ME49 cysts was 100%, while that of unvaccinated mice was only 50% (Fig. 5a). At 35 days post-infection with cysts, the parasite cyst burden in the brains of the surviving mice was assessed. The number of cysts per brain in unvaccinated mice was 1640 ± 629 cysts, whereas the cyst load per brain in ME49Δ*cdpk3-*vaccinated mice was 94 ± 21 (*p* < 0.001) (Fig. 5b). Moreover, the survival rate in ME49Δ*cdpk3*-vaccinated mice challenged with WH6 cysts was 100%, while that of unvaccinated mice was only 60% (Fig. 5c). The number of cysts per brain in unvaccinated mice was 1780 ± 572, whereas that in ME49Δ*cdpk3-*vaccinated mice was 220 ± 130 (*p* < 0.001) (Fig. 5d). These results indicate that the ME49Δ*cdpk3* strain induces a protective immune response against chronic infection with various bradyzoites.

### ME49Δ*cdpk3* vaccination induces cellular immune response

**Fig. 6:**
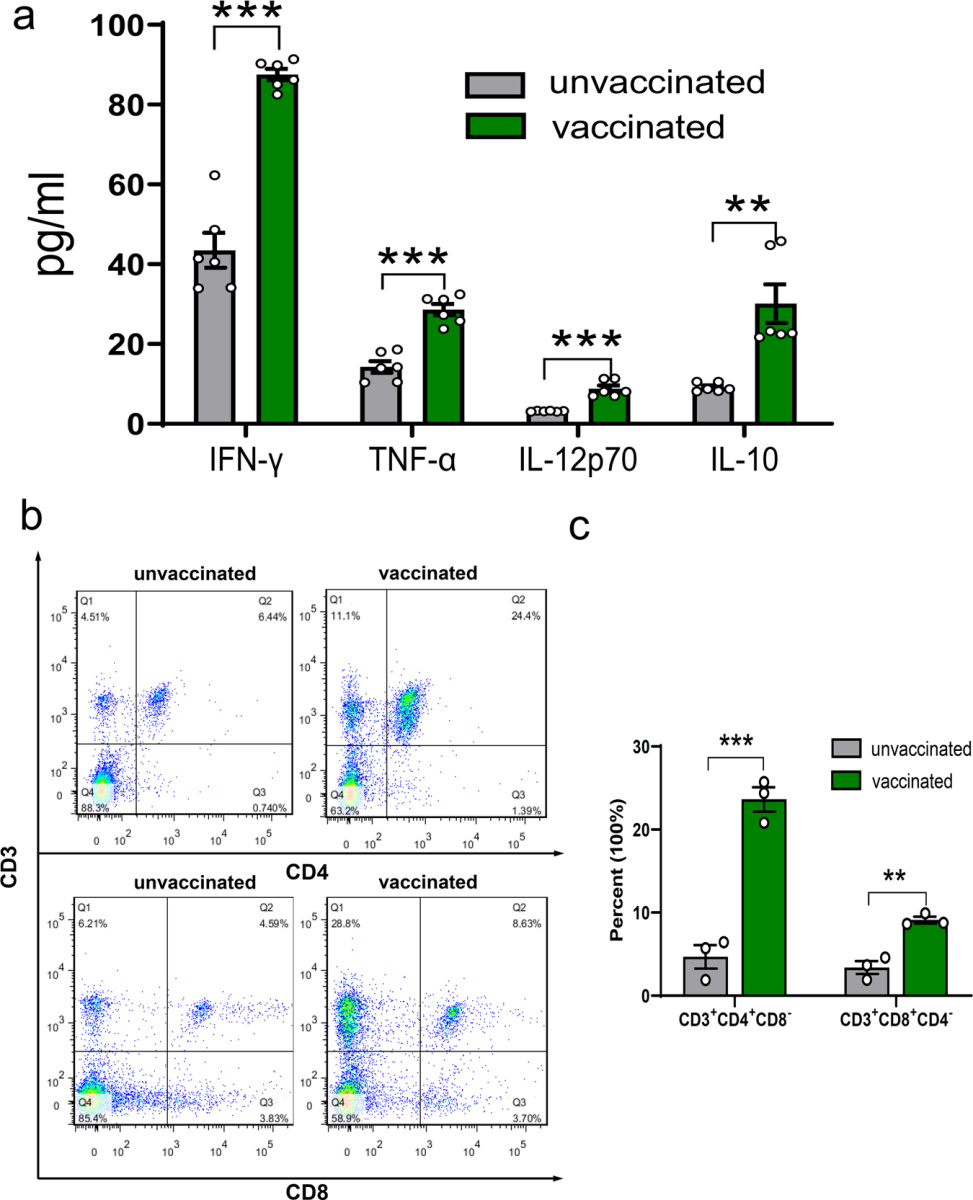
ME49Δ*cdpk3* vaccination induced cellular immune response after *Toxoplasma* antigen stimulation. **a** Spleen cells were harvested 75 days after ME49Δ*cdpk3* vaccination and stimulated in vitro with 10 µg/mL soluble ME49 antigen. IFN-γ, TNF-α, IL-12p70 and IL-10 levels in splenocyte culture supernatants were subsequently tested by ELISA (n = 6 each group). The statistical analysis was performed by using unpaired t test. Bars = mean ± SEM. Statistical differences are represented by ***p < 0.001, **p < 0.01. **b** The percentages of CD3^+^ CD8^+^ T lymphocytes and CD3^+^CD4^+^ T lymphocytes in mice spleen cells was detected by flow cytometry. Splenocytes from non-immunized mice were used as negative controls. **c** Quantitative data of (**b**) are expressed as the mean ± SEM (*n* = 3). The statistical analysis was performed by using unpaired t test. Statistical differences are represented by ***p < 0.001, **p < 0.01.

To detect the production and types of cytokines, splenocytes from ME49Δ*cdpk3*-vaccinated and unvaccinated mice were isolated at 75 dpv to assess the cell-mediated immune response in ME49Δ*cdpk3*-vaccinated animals. The splenocytes were cultured in vitro and stimulated with *T. gondii* ME49 soluble antigen. The supernatant was collected to estimate cytokine levels by ELISA. The splenocytes obtained from ME49Δ*cdpk3*-vaccinated mice had higher levels of IFN-γ, TNF-α, IL-12p70, and IL-10 compared with splenocytes obtained from nonvaccinated mice (Fig. 6a). The percentages of CD4^+ ^and CD8^+^ T cells in the spleen of ME49Δ*cdpk3*-vaccinated mice were analyzed by flow cytometry. ME49Δ*cdpk3*-vaccinated mice showed a significant increase in the percentages of CD3^+ ^CD8^+^ T cells and CD3^+^ CD4^+^ T cells compared with unvaccinated mice (Fig. 6b, c, and Supplementary Fig. 5). These results indicate that ME49Δ*cdpk3* immunization can activate efficient cellular immune responses.

### ME49Δ*cdpk3* vaccination induces a full t*oxoplasma*-specific immune response

**Fig. 7:**
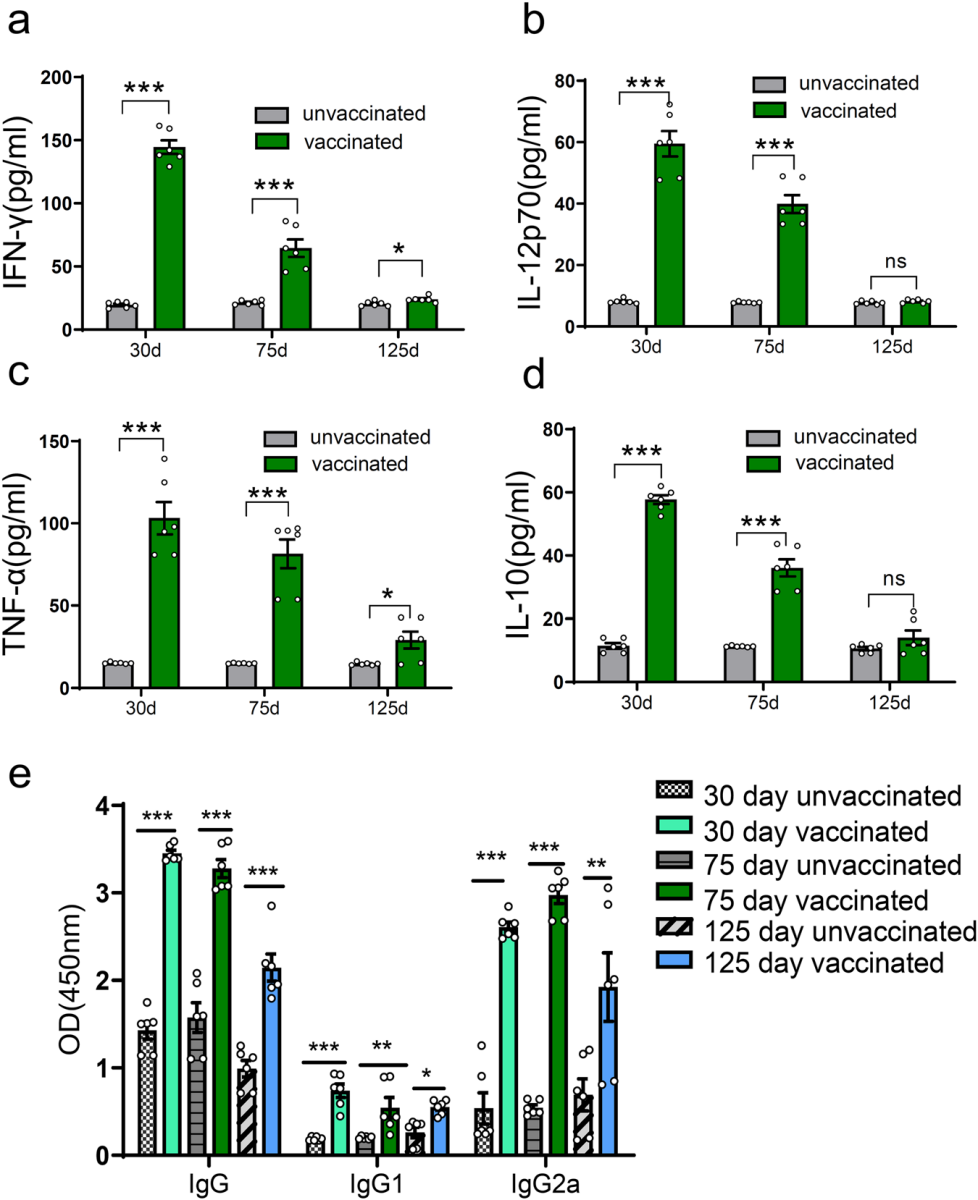
ME49Δ*cdpk3* vaccination induces full *Toxoplasma*-specific immune Response. Cytokine and *Toxoplasma*-specific IgG levels were determined by ELISA in mouse serum 30-, 75- and 125- days after immunization with ME49Δ*cdpk3*. **a** IFN-γ, **b** IL-12p70, **c** TNF-α, **d** IL-10, **e** *Toxoplasma*-specific total IgG and IgG subclass (IgG1 and IgG2a). Serum samples of unimmunized mice were considered as negative control(*n* = 6 each group). The statistical analysis was performed by using unpaired *t* test or a one-way ANOVA analysis. Bars = mean ± SEM. Statistical differences are represented by ***p < 0.001, **p < 0.01, *p < 0.05, ns not significant.

To elucidate the mechanisms of the immune response offered by ME49Δ*cdpk3* vaccination, sera obtained from mice at 30, 75, or 125 dpv were used to assess the levels of IFN-γ, IL-12p70, TNF-α and IL-10. The levels of the proinflammatory cytokines IFN-γ, IL-12p70, TNF-α and IL-10 were significantly elevated at 30, 75 and 125 dpv compared with those in unimmunized mice (Fig. 7a–d). However, cytokine levels at 75 dpv were lower than those at 30 dpv, which may be due to the activation of the anti-inflammatory response, as evidenced by the increased levels of IL-10 in mice vaccinated 30 days after infection. At 125 dpv, the cytokine levels in vaccinated mice were comparable to those of unvaccinated mice (*ns. or p* < 0.05). Next, we assessed the *T. gondii*-specific IgG levels and observed that ME49Δ*cdpk3* vaccination elicited increased levels of IgG at Days 30, 75, and 125 dpv. The immune response type triggered by ME49Δ*cdpk3* was tested by detecting the levels of IgG subclasses (IgG1 and IgG2a). Mice vaccinated with ME49Δ*cdpk3* had high levels of IgG1 and IgG2a antibodies at 30, 75, and 125 dpv, and the IgG2a titer was significantly higher than that of IgG1, suggesting that ME49Δ*cdpk3* induces a Th1-biased immune response. The results showed that the stable levels of parasite-specific IgG lasted for a relatively long time (Fig. 7e). Thus, these data indicate that ME49Δ*cdpk3* vaccination can activate both cellular and humoral immune responses to control *T. gondii* infections.

### Protective immunity with the sera of ME49Δ*cdpk3*-vaccinated mice against *T. gondii* infection

**Fig. 8:**
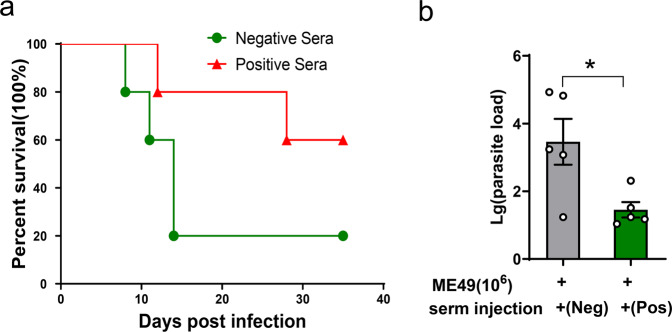
Protective immunity with the sera of ME49Δ*cdpk3*-vaccinated mice against *T. gondii* infection. Passive immunization with serum from ME49Δ*cdpk3*-vaccinated mice reduced parasite reproduction in mice. *BALB/c* mice were challenge with 10^6^ tachyzoites of ME49. At 0- and 3-day post-infection, the positive sera of ME49Δ*cdpk3*-vaccinated mice (125 days postvaccination) was injected into infected mice by tail intravenous (*n* = 10 each group). Parasite burdens in peritoneal fluids at 7 dpi were estimated by quantitative PCR. **a** Survival rates of mice. **b** Parasite burdens. The statistical analysis was performed by using unpaired t test. Bars = mean ± SEM. Statistical differences are represented by *p < 0.05.

Our previous results showed that mice vaccinated with ME49Δ*cdpk3* had high levels of *T. gondii*-specific IgG. To determine the contribution of this antibody to further limiting parasitic infections, naive mice were challenged with the 10^6^ ME49 strain by intraperitoneal injection. At 0 and 3 days postinfection, the positive sera of ME49Δ*cdpk3*-vaccinated mice (125 days postvaccination) were injected into infected mice *via* the tail vein. The passive immunization was determined by recording the survival rate of mice and the parasite load in the peritoneal fluid at 7 days postinfection. Our data showed that mice passively immunized with ME49Δ*cdpk3*-vaccinated sera had a 60% survival rate (Fig. 8a) and a significantly lower parasite load than unimmunized mice. (Fig. 8b). Altogether, these results suggested that the sera of mice vaccinated with ME49Δ*cdpk3* can reduce the proliferation of parasites to a certain extent.

## Discussion

Toxoplasmosis is a zoonotic disease that causes great public health concern. It not only poses a serious threat to humans but also causes great economic losses to the animal industry. Therefore, it is necessary to discover and develop a highly effective vaccine against toxoplasmosis. Ca^2+^-dependent protein kinases^17,31,32^, microneme proteins^33^, rhoptry proteins^15,20^, and dense granule proteins^34,35^ have been studied for their ability to induce protective immune responses against acute and latent *Toxoplasma* infections in mice. These nucleic acid vaccines, recombinant protein vaccines and mixed cocktail vaccines are safer and easier to manufacture than vaccines based on native antigens. However, they do not provide long-term protection against a variety of wild-type strains^36,37^. Live-attenuated vaccines seem to be the most promising strategy for long-term protective effects^22,23,32^. At present, Toxovax^®^ is the only approved live attenuated vaccine for use in sheep and goats to prevent abortion^24^. In the present study, ME49Δ*cdpk3* live-attenuated vaccination elicited a strong immunological response and protected mice from acute and latent infection with a variety of *Toxoplasma* strains.

CDPK3 is essential for the rapid induction of parasite egress and the virulence of the parasite^29,38^. Current results demonstrate that CDPK3-deficient parasite strains had decreased virulence and cyst formation in mice^4,30^. In this study, we used 10^3^, 10^4^, 10^5^, 10^6^ wild-type ME49 and ME49Δ*cdpk3* tachyzoites to vaccinate mice and monitor the survival rate of the mice. At the immunization dose of 10^6^ tachyzoites/mouse, the survival rate of ME49Δ*cdpk3*-vaccinated mice was still 100% at 35 dpi (Fig. 2a). To obtain the appropriate immunogenicity needed for the attenuated ME49Δ*cdpk3* strain without generating an excessive immune response in the vaccinated mice, we administered 10^3^ ME49Δ*cdpk3* tachyzoites per mouse. We found that mice vaccinated with ME49Δ*cdpk3* were efficiently protected against a variety of wild-type strains, including type I RH, type II ME49, Chinese 1 WH3 and Chinese 1 WH6 infections. ME49Δ*cdpk3* vaccination elicited a robust immune response when challenged with different strains. Notably, the parasite load in the peritoneal fluid was greatly reduced in ME49Δ*cdpk3*-vaccinated mice challenged with RH, ME49, WH3 or WH6 tachyzoites (*p* < 0.001 or *p* < 0.01) (Fig. 3c and f). Remarkably, the survival rate was 100% in vaccinated mice infected with the less virulent ME49 and WH6 strains (10^5^ tachyzoites), while the survival rate was approximately 20% in unvaccinated mice. Although the survival times of infection with the virulent RH or WH3 tachyzoites were not high, the survival rates were significantly extended (Fig. 3b and e). In addition, ME49Δ*cdpk3* attenuated the development of chronic toxoplasmosis in mice. Compared with unvaccinated mice, the survival rate of the vaccinated mice infected with 50 ME49 or WH6 cysts was 100% (Fig. 3a and d), and the numbers of parasite cysts in the brain were significantly reduced (Fig. 5). *T. gondii* consists of three main genotypes in North America and Europe, designated types I, II, and III, and they vary in virulence. The type I genotype is uniformly lethal in all strains of laboratory mice, even with a low inoculum, and is accurately referred to as virulent. The type III genotype causes little or no mortality in all mouse strains and is referred to as avirulent. The type II genotype has intermediate virulence, which can be expressed as the dose that causes 50% lethality (LD_50_). This strain is most commonly associated with human infections in both cases of congenital infection^39^ and immunocompromised patients^40^, and this pattern is also seen in North America and Europe. Therefore, ME49Δ*cdpk3* vaccination might be safe and highly effective for the majority of human infections arising from less virulent strains.

To evaluate the potential protective immunity of ME49Δ*cdpk3* vaccination, the levels of cytokines and antibodies in mice were detected. *T. gondii* IgG levels remained high and stable during the entire vaccination period. While testing IgG subclasses, we found that the levels of IgG1 and IgG2a in immunized mice were significantly elevated, with a greater increase in IgG2a, compared with unimmunized mice (Fig. 7e). These results indicated that ME49Δ*cdpk3* vaccination triggers both Th1 and Th2 immune responses, with the Th1 response being dominant. Previous studies have demonstrated that a Th1-biased immune response can be effective for preventing *Toxoplasma gondii* infection^17,41,42^. The proinflammatory cytokines IFN-γ, IL-12 and TNF-α are essential for the activation of the cell-mediated immune response against *Toxoplasma* infection^43,44^. IFN-γ can regulate various intracellular mechanisms to kill parasites and inhibit their replication and is the main mediator of resistance to *T. gondii*^43^. The inflammatory factor IL-12 can stimulate IFN-γ release from CD4^+^ and CD8^+^ T cells and natural killer (NK) cells^45^. TNF-α is required to provide additional signals to synergize with IFN-γ to kill parasites^46^. The anti-inflammatory cytokine IL-10 is responsible for the downregulation of IFN-γ during the acute stage of immune responses^47,48^. At 30 days post-vaccination, serum levels of IFN-γ, TNF-α, IL-12p70 and IL-10 were significantly elevated in vaccinated mice compared with unvaccinated mice and returned to normal levels at 125 days post-vaccination (Fig. 7a–d), indicating that ME49Δ*cdpk3* vaccination maintained a balanced cytokine level. The high levels of the proinflammatory cytokines IFN-γ, IL-12p70 and TNF-α and the anti-inflammatory cytokine IL-10 in the splenocyte stimulation test also confirmed this balance (Fig. 6a). More importantly, our study demonstrated that the high levels of *Toxoplasma*-specific antibodies elicited by the ME49Δ*cdpk3* vaccine were maintained for 125 days, a relatively long time compared with previous studies^21,22,49^. Our study of serum passive transfer also showed that the ME49Δ*cdpk3* vaccine conferred significant long-term protection against *Toxoplasma* infection (Fig. 8).

CD4^+^ T cells are essential for resistance to toxoplasmosis. During the early stages of infection, the parasite can promote B and CD8^+^ T cells to produce IFN-γ. CD4^+^ T cells can also provide several key regulatory functions to mediate resistance to toxoplasmosis^41,50^. CD8^+^ T cells play a key role in recognizing and destroying cells infected by viruses, bacteria and parasites. It acts mainly through the following three methods: through the production of inflammatory cytokines, such as IFN-γ, through the CD40/CD40 L interaction, and through the cytolysis of infected host cells mediated by perforin^41,51,52^. Flow cytometry analysis showed that CD8^+^ and CD4^+^ cytotoxic T lymphocytes were activated in the immunized mice (Fig. 6b). When rechallenged with wild-type *T. gondii* parasites, cytokine levels in vaccinated mice were lower than those in unvaccinated mice, indicating that a severe immune response was not evoked in the vaccinated mice of the second challenge (Fig. 4a–d). However, *Toxoplasma*-specific IgG was maintained at a high level to effectively eliminate secondary *Toxoplasma* infections (Fig. 4e). Passive immunization tests suggested that serum from mice vaccinated with the ME49Δ*cdpk3* vaccine can significantly limit the proliferation of parasites (Fig. 8). These results showed that ME49Δ*cdpk3* vaccination induced effective humoral and cellular immunity against *T. gondii* infection. However, the current study has some limitations. First, one limitation of this study is that it was only based on a mouse model. Testing of the effectiveness and safety of the ME49Δ*cdpk3* vaccine should be expanded to animals of veterinary and economic importance, including but not limited to definitive hosts cats, susceptible sheep and pigs and other meat-producing animals. Second, ME49*Δcdpk3* vaccination produced a very small amount of tissue cysts, although these cysts were severely attenuated and likely would not cause diseases in vaccinated animals. One strategy to decrease the amount of tissue cysts formed after vaccination is to develop a live vaccine with multiple deletions in one strain. Finally, the ME49*Δcdpk3* still need to assess its effectiveness against other *Toxoplasma* strains, particularly infections with local endemic strains.

In conclusion, our study showed that the depletion of CDPK3 in the ME49 strain significantly attenuated the virulence of parasites and dramatically reduced the formation of cysts in mice. ME49Δ*cdpk3* vaccination elicits both cellular and humoral immunity and protects mice from infection by a variety of wild-type *Toxoplasma* strains, including type I RH, type II ME49, Chinese 1 WH3 and Chinese 1 WH6. This finding indicates that the ME49Δ*cdpk3* strain could be a viable live-attenuated vaccine candidate against acute and latent toxoplasmosis.

## Methods

### Animals and parasites

Female *BALB/c* mice aged 6–8 weeks were cohoused under specific pathogen-free standard conditions and had free access to sterilized water and food. All experimental procedures were approved by the Scientific Ethics Committee of Anhui Medical University (permit number: LLSC20200036). All experimental procedures were performed in strict accordance with the recommendations in the Guide for the Care and Use of Laboratory Animals of Anhui Medical University. All efforts were made to lighten the suffering of these research animals. *Toxoplasma gondii* type I strain RH, type II strain ME49, ME49Δ*cdpk3*, Chinese1 strain WH3 and Chinese 1 strain WH6 were used in this study. All parasites were maintained in human foreskin fibroblast cells (purchased from ATCC, USA). The *Toxoplasma* ME49 strain bradyzoites were maintained through oral passage of cysts in Kunming mice.

### Generation of the ME49Δ*cdpk3* strain by CRISPR/Cas9

The CDPK3 gene in ME49 was knocked out by CRISPR/Cas9 gene editing technology as described previously^53^. The primers and plasmids used for gene editing are listed in Supplementary Table 1.

### Virulence tests of the WT strain versus the ME49Δ*cdpk3* strain in mice

*BALB/c* mice aged 7 weeks (10 mice per group) were infected with freshly egressed tachyzoites by intraperitoneal injection (i.p.). The survival rate of the infected mice was monitored over 35 days. Sera in peripheral blood were obtained from surviving mice on Day 30 *via* tail vein sampling. At 7 days postinfection (dpi), the brain, eyes, liver, ileum, spleen, heart, and blood were obtained from infected mice, and genomic DNA was extracted using the SteadyPure Genomic DNA Kit (AG Biotech, Hunan, China). Parasite burdens in the brain, eyes, liver, ileum, spleen, heart, and blood were determined by qPCR.

### Protection against acute and chronic infection

*BALB/c* mice were immunized with 10^3^ ME49Δ*cdpk3* tachyzoites by intraperitoneal injection. At 75 days after immunization, mice were infected with 1 × 10^3^ Chinese 1 WH3 tachyzoites by injection, 1 × 10^5^ Chinese 1 WH6 tachyzoites by injection, or 20 ME49 cysts by oral administration (*n* = 10 per group). At 125 days after immunization, mice were injected with 1 × 10^3^ RH and 1 × 10^5^ ME49 tachyzoites (*n* = 10 per group). Unvaccinated mice infected with the same dose by the same route served as controls. These reinfected mice were then monitored daily for an additional 35 days for clinical symptoms and survival. At 7 dpi, the peritoneal fluid of mice was collected to measure cytokine production by ELISA and determine the parasite burden (6 mice/group). For chronic infections, the number of cysts in the brains of surviving mice was detected on Day 35 after challenge (5 mice/group).

### Detection of cytokines and t*oxoplasma*-specific IgG level

*BALB/c* mice were immunized with 1×10^3^ ME49Δ*cdpk3* tachyzoites or mock-vaccinated with 300 μl PBS i.p. Serum samples were obtained at 30, 75 and 125 days postvaccination (dpv). Levels of total *Toxoplasma*-specific IgG and IgG subclasses (IgG1 and IgG2a) were measured by enzyme-linked immunosorbent assay (ELISA). Briefly, 10 μg/ml soluble ME49 antigens diluted in coating buffer (50 mM carbonate buffer, pH 9.6) were used to coat 96-well ELISA plates. Then, the plates were incubated overnight at 4 °C and washed with phosphate buffer saline containing 0.05% Tween-20 (PBST, pH 7.4). Then, 3% BSA was used to block nonspecific binding for 1 h at 37 °C, and the plates were washed five times with PBST. The collected serum was diluted 1:50 and incubated at 37 °C for 1 h. After washing, HRP-conjugated goat anti-mouse IgG and subclasses IgG1 or IgG2a (ProteinTech Group, Inc., USA) diluted in PBST (1:1000) were added to each well (100 μl per well), and the plates were incubated for an additional 1 h at 37 °C. Then, each well was washed 5 times with PBST. Finally, TMB (100 μl/well, Beyotime Biotechnology) was used as a substrate to develop the reaction. Then, 2 M H_2_SO_4_ was added to stop the reaction. The optical density (OD) was measured with an ELISA reader at 450 nm. All serum samples were analyzed in triplicate. Meanwhile, the production of the cytokines IFN-γ, IL-12p70, TNF-α and IL-10 was measured using ELISA kits according to the manufacturer’s recommendations.

### Cytokine production in splenocyte supernatants

Both immunized and unimmunized mice were anesthetized and sacrificed, and spleens were harvested to assess the levels of cytokine production. Briefly, splenocytes were isolated through a 70 μM wire mesh sieve and hemolyzed in lysing buffer (BD, United States) for 5 min to obtain a single spleen cell suspension. Splenocytes were cultured in 24-well plates and stimulated with 10 µg/mL *T. gondii* soluble tachyzoite antigen (STAg) of the ME49 strain. Cell-free supernatants were then collected to measure the levels of TNF-α and interleukin 10 (IL-10) at 72 h post-incubation and interleukin 12p70 (IL-12p70) and interferon-gamma (IFN-γ) at 96 h postincubation. Unvaccinated mouse splenocytes were used as negative controls.

### Flow cytometry analysis of lymphocytes

To analyze the percentage of CD4^+ ^and CD8^+^ T lymphocytes, splenocytes were prepared as described above, and 1×10^6^ cells were suspended in 100 µl PBS. After incubation with fluorochrome-labeled mAbs, including APC-CD3, FITC-CD4 and PE-CD8 (BioLegend, United States), at 4 °C for 30 min in the dark, the cell suspension was washed twice with 1 ml PBS and then fixed with FACScan buffer (PBS containing 1% FCS and 0.1% sodium azide). All samples were analyzed by FCM (BD, United States).

### Passive immunization with the sera of ME49Δ*cdpk3*-vaccinated mice

*BALB/c* mice were infected with 1×10^6^ type II ME49 tachyzoites through intraperitoneal injection. Sera from naive mice or immunized mice at 125 dpv were injected into infected mice via the tail vein (100 μl/mouse) at 0 and 3 days postchallenge. Naive sera served as a negative control. Parasite burden in peritoneal fluids was examined at 7 days postchallenge by quantitative PCR to measure parasite proliferation under passive immunization. The number of deaths in each group of the remaining mice was recorded daily for 35 days.

### DNA isolation and determination of parasite burden in challenged mice

Genomic DNA was extracted from the peritoneal fluid of parasite-infected mice using the Steady Pure Genomic DNA Kit (AG Biotech, Hunan, China). Parasite burden was determined by qPCR amplification of the Tg-529 gene (forward primer 5’- CGCTGCAGGGAGGAAGACGAAAGTTG-3’ and reverse primer 5’- CGCTGCAGACAGAGTGCATCTGGATT-3’) using corresponding genomic DNA samples. The values were normalized to the number of mouse-actin genes (forward primer 5’-AGCTTCTTTGCAGCTCCTTCGT-3’ and reverse primer 5’- TACACGCTAGGCGTAAAGTTGG-3’) in each sample. Real-time qPCR was carried out with the SYBR^®^ Premix Ex TaqTM II Kit (TaKaRa, Dalian, China) according to the manufacturer’s instructions, and reactions were run on the Roche LC480II system. A standard curve was obtained for the quantification of parasites. Genomic DNA from 0, 10^0^, 10^1^, 10^2^, 10^3^, 10^4^, 10^5^, 10^6^ and 10^7^ parasites were extracted. The CT value of each sample was then obtained using Tg529-based qPCR as the ordinate and Lg (tachyzoite number) as the abscissa. Subsequently, a standard curve was generated from the CT values and Lg, and the parasite burden was calculated from the standard.

### Statistical analysis

Statistical analysis was performed and graphics were generated in GraphPad Prism 8.0 (GraphPad Software Inc., La Jolla, CA, USA). In this study, unpaired t test was applied to compare two groups, and a one-way ANOVA was applied for the comparison of multiple groups. Gehan–Breslow–Wilcoxon test was used for survival comparison. A value of *p* < 0.05 was considered statistically significant.

### Reporting summary

Further information on research design is available in the Nature Research Reporting Summary linked to this article.

## Supplementary information

Supplementary information

REPORTING SUMMARY

## Data Availability

The original data supporting the conclusions of this article will be provided by the authors without undue retention.
