# Supplementary material for: Live-attenuated ME49Δcdpk3 strain of Toxoplasma gondii protects against acute and chronic toxoplasmosis
Source: NPJ Vaccines. 2022 Aug 20;7:98. doi: 10.1038/s41541-022-00518-5 (PMC9391373; doi:10.1038/s41541-022-00518-5)
Supplement: Supplementary file 1 — Supplementary information [file 41541_2022_518_MOESM1_ESM.pdf]

SUPPLEMENTARY INFORMATION

Supplementary Table1. The primers and plasmids used in CRISPR/Cas9 gene editing technology are listed.

Primers	Sequence	Used for
CDPK3-gRNA-F<br>CDPK3-gRNA-R	TGTACCGAGGGTTTTAGAGCTAGAAATAGC<br>CCTCCATGACAACTTGACATCCCCATTTAC	Q5 mutagenesis changing the gRNA in pSAG1:CAS9-U6:sgUPRT to gRNA-CDPK3
UpCDPK3-F<br>UpCDPK3-R	AAAACGACGGCCAGTGAATTCAGCCAACATGCATTGGAGCT<br>GGGGGTGAAAATCGAATGACAACGCAGCGACTGGGAGAATC	To produce UpCDPK3 PCR product for making pCDPK3: DHFR*-TS
DHFR*-TS -F<br>DHFR*-TS -R	TGTCATTCGATTTTCACCCCC<br>AGTGTGATGACTCCGCAACTGGATCGATCCCCCCGGGCTGC	To produce DHFR*-TS PCR product for making pCDPK3: DHFR*-TS
DnCDPK3-F<br>DnCDPK3-R	GCAGCCCGGGGGGATCGATCCAGTTGCGGAGTCATCACACT<br>GACCATGATTACGCCAAGCTTTCGTTGTGGGCTATACAGCT	To produce DnCDPK3 PCR product for making pCDPK3: DHFR*-TS
PCR1-F<br>PCR1-R	GCCTAACAAGGATTCGATCAGTAGC<br>TGTCGTGGATTTACCAGTCATGGAC	To examine the integration of DHFR*-TS into corresponding genes
PCR2-F<br>PCR2-R	TGACTCTTCATGTGGCATTTCACAC<br>TACTGTGTTAGGTAGCAAATGTGG	To examine the integration of DHFR*-TS into corresponding genes
PCR3-F<br>PCR3-R	TCGTGCGTCTTCAGGCATGTACATC<br>GAGGTCCTTTCGCTTCCTGAGACTC	To examine the deletion of CDPK3 sequences

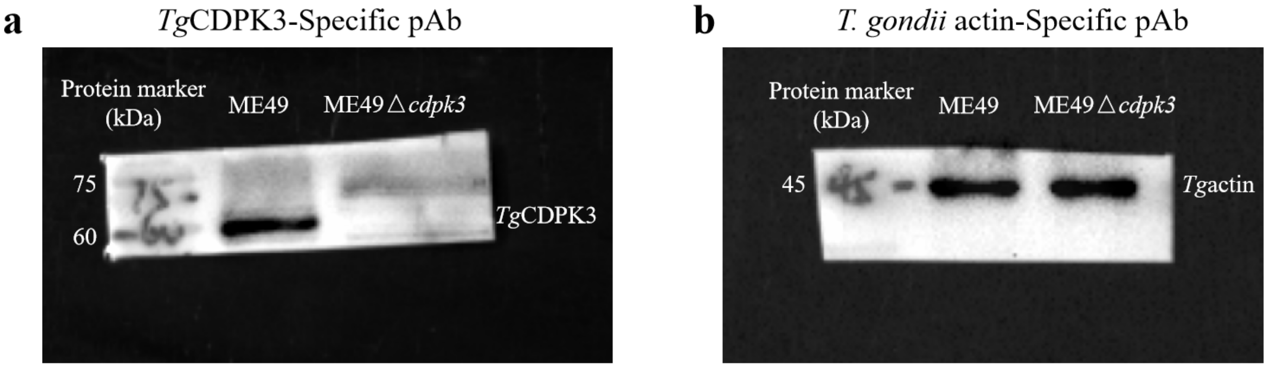

Supplementary Figure 1. Western blot analysis.
All the western blots were derived from the same experiment and processed in parallel. (a) TgCDPK3 specific western blot against proteins extracted from the indicated strains. (b) *T. gondii* actin specific western blot against proteins extracted from the indicated strains.

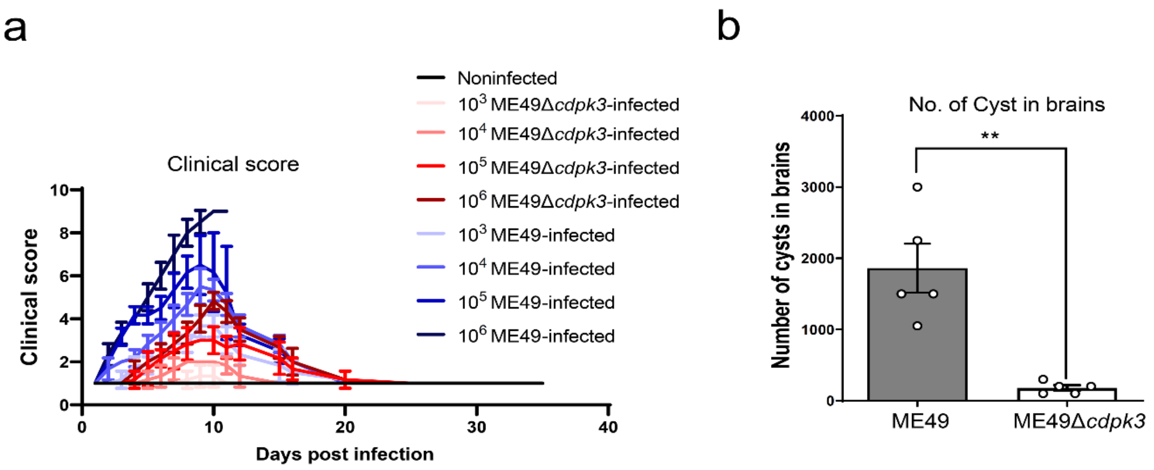

Supplementary Figure 2. Clinical symptoms of mice during parasite infection.
(a)The different dose of 10^3, 10^4, 10^5 and 10^6 ME49Δ*cdpk3* or 10^3, 10^4, 10^5 and 10^6 wild type ME49 were injected into mice, and clinical signs of infected mice were noted within 35 days post-infection. The clinical scores varied from 0 (no signs) to 10 (all signs). No-infected mice were used as control. (b) The number of cysts in the brains of ME49Δ*cdpk3*-, or wild type ME49-infected mice. Each mouse was injected with 10^5 ME49Δ*cdpk3* or wild type ME49 tachyzoites and the number of cysts in mouse brains was calculated 35 days after infection (n=5 each group). The statistical analysis was performed using by unpaired t test. Bars = mean ± SEM. **$p < 0.01$.

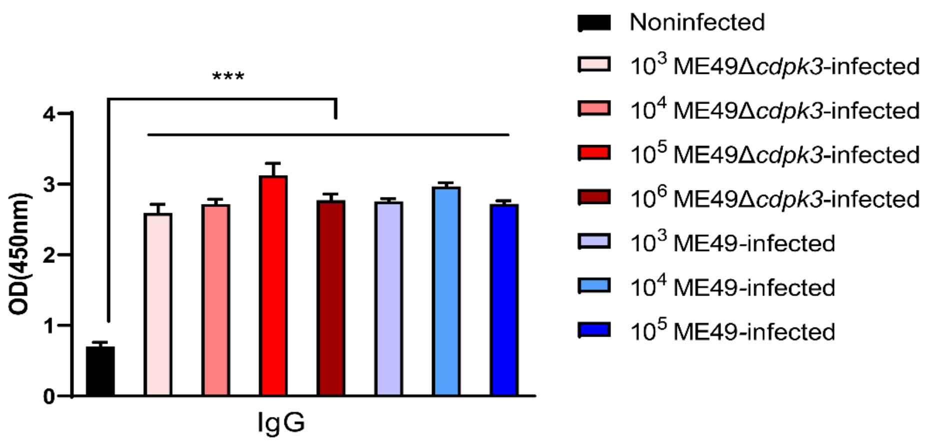

Supplementary Figure 3. Different doses of 10^3, 10^4, 10^5, and 10^6 ME49Δ*cdpk3,* wild type ME49 tachyzoites were injected into mice, respectively, and *Toxoplasma*-specific IgG levels were detected on day 35 post-infection (n=6 each group). The statistical analysis was performed using by one-way ANOVA analysis. Bars = mean ± SEM. ***$p < 0.001$.

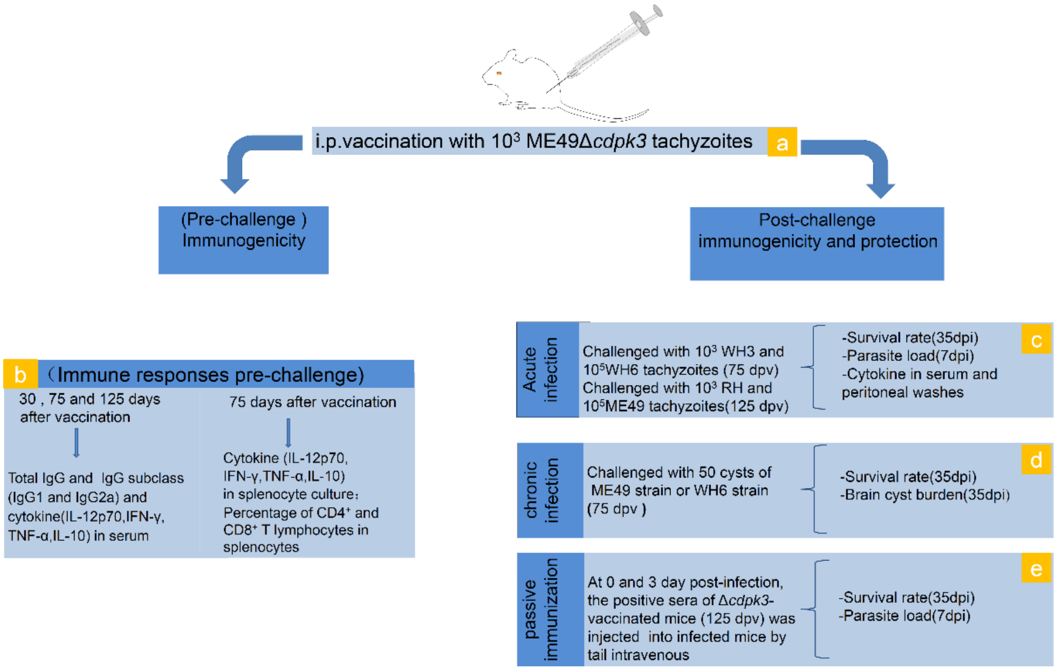

Supplementary Figure 4. Schematic diagram of vaccination study design.
(a) Experimental overview of this study immunized with 1×10^3 ME49Δ*cdpk3* tachyzoites in *BALB/c* mice (b) to assess the immune response in the serum and splenocytes of immunized mice prior to infection (c) to assess the efficacy of immunization against acute infection (d)and chronic infection (e)To assess passive immunization with the sera of ME49Δ*cdpk3*-vaccinated mice resist *T. gondii* infection. Abbreviations: i.p., intraperitoneal; dpv, days post-vaccination; dpi, days post-infection.

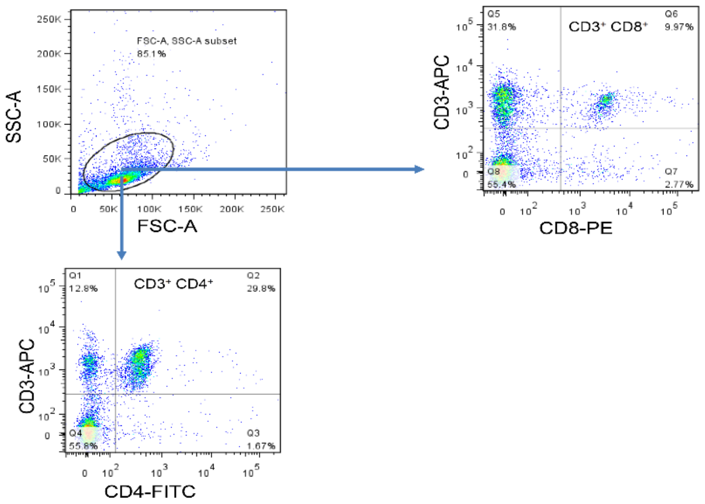

Supplementary Figure 5. Gating strategies used for splenocytes analysis.
Gating strategies used for analyzing the percentage of CD3$^+$CD4$^+$ and CD3$^+$CD8$^+$ T lymphocytes from unvaccinated and vaccinated *BALB/c* mice presented on Figure 6 b and c.
