# Supplementary material for: Live-attenuated ME49Δcdpk3 strain of Toxoplasma gondii protects against acute and chronic toxoplasmosis
Source: NPJ Vaccines. 2022 Aug 20;7:98. doi: 10.1038/s41541-022-00518-5 (PMC9391373; doi:10.1038/s41541-022-00518-5)
Supplement: Supplementary file 2 — REPORTING SUMMARY [file 41541_2022_518_MOESM2_ESM.pdf]

nature portfolio

Corresponding author(s): Jian Du

Last updated by author(s): Jun 11, 2022

Reporting Summary

Nature Portfolio wishes to improve the reproducibility of the work that we publish. This form provides structure for consistency and transparency in reporting. For further information on Nature Portfolio policies, see our Editorial Policies and the Editorial Policy Checklist.

Statistics

For all statistical analyses, confirm that the following items are present in the figure legend, table legend, main text, or Methods section.

n/a	Confirmed	
☐	☒	The exact sample size (*n*) for each experimental group/condition, given as a discrete number and unit of measurement
☐	☒	A statement on whether measurements were taken from distinct samples or whether the same sample was measured repeatedly
☐	☒	The statistical test(s) used AND whether they are one- or two-sided<br>*Only common tests should be described solely by name; describe more complex techniques in the Methods section.*
☒	☐	A description of all covariates tested
☒	☐	A description of any assumptions or corrections, such as tests of normality and adjustment for multiple comparisons
☐	☒	A full description of the statistical parameters including central tendency (e.g. means) or other basic estimates (e.g. regression coefficient) AND variation (e.g. standard deviation) or associated estimates of uncertainty (e.g. confidence intervals)
☐	☒	For null hypothesis testing, the test statistic (e.g. F, t, r) with confidence intervals, effect sizes, degrees of freedom and P value noted<br>*Give P values as exact values whenever suitable.*
☒	☐	For Bayesian analysis, information on the choice of priors and Markov chain Monte Carlo settings
☒	☐	For hierarchical and complex designs, identification of the appropriate level for tests and full reporting of outcomes
☒	☐	Estimates of effect sizes (e.g. Cohen's *d*, Pearson's *r*), indicating how they were calculated

Our web collection on statistics for biologists contains articles on many of the points above.

Software and code

Policy information about availability of computer code

Data collection	Microsoft Excel, FlowJo
Data analysis	GraphPad Prism 8

For manuscripts utilizing custom algorithms or software that are central to the research but not yet described in published literature, software must be made available to editors and reviewers. We strongly encourage code deposition in a community repository (e.g. GitHub). See the Nature Portfolio guidelines for submitting code & software for further information.

Data

Policy information about availability of data

All manuscripts must include a data availability statement. This statement should provide the following information, where applicable:

- Accession codes, unique identifiers, or web links for publicly available datasets
- A description of any restrictions on data availability
- For clinical datasets or third party data, please ensure that the statement adheres to our policy

The original data supporting the conclusions of this article will be provided by the authors without undue retention.

Human research participants

Policy information about studies involving human research participants and Sex and Gender in Research.

Reporting on sex and gender	*Use the terms sex (biological attribute) and gender (shaped by social and cultural circumstances) carefully in order to avoid confusing both terms. Indicate if findings apply to only one sex or gender; describe whether sex and gender were considered in study design whether sex and/or gender was determined based on self-reporting or assigned and methods used. Provide in the source data disaggregated sex and gender data where this information has been collected, and consent has been obtained for sharing of individual-level data; provide overall numbers in this Reporting Summary. Please state if this information has not been collected. Report sex- and gender-based analyses where performed, justify reasons for lack of sex- and gender-based analysis.*
Population characteristics	*Describe the covariate-relevant population characteristics of the human research participants (e.g. age, genotypic information, past and current diagnosis and treatment categories). If you filled out the behavioural & social sciences study design questions and have nothing to add here, write "See above."*
Recruitment	*Describe how participants were recruited. Outline any potential self-selection bias or other biases that may be present and how these are likely to impact results.*
Ethics oversight	*Identify the organization(s) that approved the study protocol.*

Note that full information on the approval of the study protocol must also be provided in the manuscript.

Field-specific reporting

Please select the one below that is the best fit for your research. If you are not sure, read the appropriate sections before making your selection.

☒ Life sciences ☐ Behavioural & social sciences ☐ Ecological, evolutionary & environmental sciences

For a reference copy of the document with all sections, see nature.com/documents/nr-reporting-summary-flat.pdf

Life sciences study design

All studies must disclose on these points even when the disclosure is negative.

Sample size	In this study,all animal experiments were performed with aged 6-8 weeks female BALB/c mice.The sample size was based on previous studies of Toxoplasma gondii vaccine.
Data exclusions	No data was excluded from any experiments.
Replication	All experiments were repeated three times and all data were reproducible.
Randomization	All experiment animals were divided into groups randomly.
Blinding	This is a study investigating the efficacy of ME49Δcdpk3 as a live attenuated vaccine against Toxoplasma gondii, and does not require blinding.

Reporting for specific materials, systems and methods

We require information from authors about some types of materials, experimental systems and methods used in many studies. Here, indicate whether each material, system or method listed is relevant to your study. If you are not sure if a list item applies to your research, read the appropriate section before selecting a response.

Materials & experimental systems

n/a	Involved in the study	
☐	☒	Antibodies
☐	☒	Eukaryotic cell lines
☒	☐	Palaeontology and archaeology
☐	☒	Animals and other organisms
☒	☐	Clinical data
☒	☐	Dual use research of concern

Methods

n/a	Involved in the study	
☒	☐	ChIP-seq
☐	☒	Flow cytometry
☒	☐	MRI-based neuroimaging

Antibodies

Antibodies used	Goat Anti-Rabbit IgG(H+L)-HRP conjugate((proteintech, China, #SA00001-2). HRP-conjugated goat anti-mouse subclasses IgG1 (proteintech, China, #SA00012-1)or IgG2a (proteintech, China, #SA00012-2).TgCDPK3 antibody was prepared by Taopu company (Taopu, shanghai). T. gondii actin antibody was kindly provided by Professor Yu Li (Anhui Medical University, China). APC-CD3(biolegend,Clone: 17A2, #100235,United States), FITC-CD4 (biolegend, Clone: RM4-5,#100509,United States)and PE-CD8 (biolegend, Clone: 53-6.7,#100707,United States)
Validation	See manufacturer's website for validation of commercial antibodies

Eukaryotic cell lines

Policy information about cell lines and Sex and Gender in Research

Cell line source(s)	human foreskin fibroblast cells (SCRC-1041)was purchased from ATCC
Authentication	N/A
Mycoplasma contamination	N/A
Commonly misidentified lines (See ICLAC register)	N/A

Animals and other research organisms

Policy information about studies involving animals; ARRIVE guidelines recommended for reporting animal research, and Sex and Gender in Research

Laboratory animals	Female BALB/c female mice aged 6-8weeks were used.
Wild animals	N/A
Reporting on sex	N/A
Field-collected samples	All BALB/C mice involved in the study were maintained under specific pathogen-free conditions
Ethics oversight	All experimental procedures were approved by the Scientific Ethic Committee of Anhui Medical University

Note that full information on the approval of the study protocol must also be provided in the manuscript.

Flow Cytometry

Plots

Confirm that:

☒ The axis labels state the marker and fluorochrome used (e.g. CD4-FITC).

☒ The axis scales are clearly visible. Include numbers along axes only for bottom left plot of group (a 'group' is an analysis of identical markers).

☒ All plots are contour plots with outliers or pseudocolor plots.

☒ A numerical value for number of cells or percentage (with statistics) is provided.

Methodology

Sample preparation	splenocytes were isolated and 1×106 cells were suspended in 100 μl PBS. After incubation with fluorochrome-labeled mAbs including APC-CD3, FITC-CD4 and PE-CD8 at 4◦C for 30 min in the dark, the cell suspension was washed twice with 1 ml PBS and then fixed with FACScan buffer (PBS containing 1% FCS and 0.1% Sodium azide).
Instrument	BD, United States
Software	FlowJo and Excel
Cell population abundance	N/A
Gating strategy	FSC/SSC for single lymphocytes were gating as targeted cells, then cells were gating as anti-CD3-APC positive, CD4+ T cells

Gating strategy

and CD8+ T cells were gated with anti-CD4-FITC and anti-CD8-PE antibodies. The percentages of CD3+CD8+ T cells and CD3+CD4+ T cells were then compared between the immunized and unimmunized groups

☒ Tick this box to confirm that a figure exemplifying the gating strategy is provided in the Supplementary Information.
